# Accuracy of self-reported BMI using objective measurement in high school students

**DOI:** 10.1017/jns.2020.28

**Published:** 2020-08-12

**Authors:** Chelsea Allison, Sarah Colby, Audrey Opoku-Acheampong, Tandalayo Kidd, Kendra Kattelmann, Melissa D. Olfert, Wenjun Zhou

**Affiliations:** 1 Department of Nutrition, University of Tennessee, 1215 W. Cumberland Avenue, Knoxville, TN 37996, USA; 2 Department of Food, Nutrition, Dietetics and Health, Kansas State University, 1324 Lovers Lane, Manhattan, KS 66506, USA; 3 Health and Nutritional Sciences Department, South Dakota State University, Wagner Hall 425, Brookings, SD 57007, USA; 4 Davis College of Agriculture, Natural Resources and Design School of Agriculture, West Virginia University, 1194 Evansdale Drive, G28 Agricultural Sciences Building, Morgantown, WV 26506, USA; 5 Department of Business Analytics and Statistics, University of Tennessee, 916 Volunteer Blvd., Knoxville, TN 37996-0532, USA

**Keywords:** Anthropometrics, Adolescents, BMI, Self-report, BMI, body mass index, CCC, concordance correlation coefficient, LOA, limits of agreement, TEM, technical error of measurement, rTEM, relative technical error of measurement

## Abstract

Self-reported measures for body mass index (BMI) are considered a limitation in research design, especially when they are a primary outcome. Studies have found some populations to be quite accurate when self-reporting BMI; however, there is mixed research on the accuracy of self-reported measurements in adolescents. The aim of this study is to examine the accuracy of self-reported BMI by comparing it with measured BMI in a sample of U.S. adolescents and to understand gender differences. This cross-sectional study collected self-reported height and weight measurements of students from five high schools in four states (Tennessee, South Dakota, Kansas and Florida). Trained researchers took height and weight of students for an objective measurement. BMI was calculated from both sources and categorized (underweight, normal, overweight and obese) using the Centers for Disease Control and Prevention's BMI-for-age percentiles. Participants (*n* 425; 51⋅0 % female) had a mean age of 16⋅3 years old, and the majority were White (47⋅5 %). Limits of agreement (LOA) analysis revealed that BMI and weight were underreported, and height was overreported in the overall sample, in females, and in males. LOA analysis was fair for BMI in all three groups. Overall agreement in BMI categorisation was considered substantial (*Κ* 0⋅71, *P* < 0⋅001). As BMI increased, more height and weight inaccuracies led to decreased accuracy in BMI categorisation, and the specificity of obese participants was low (50⋅0 %). This study's findings suggest that using self-reported values to categorize BMI is more accurate than using continuous BMI values when self-reported measures are used in health-related interventions.

## Introduction

Almost 19 % of youth (2–19 years old) were obese in 2015–2016, and 14⋅8 % of high school students (grades 9–12) were obese in 2017^(1,2)^. Childhood obesity can lead to negative psychosocial, neurological, pulmonary, cardiovascular, gastrointestinal, endocrine, renal and musculoskeletal consequences^(3)^. Obese children can be stigmatised by peers, family and other adults in their life, and often have a lower quality of life than peers of a healthy weight^(4)^. Obesity can result in the early onset of puberty in girls^(5)^, and overweight and obesity are associated with poor levels of academic achievement^(6)^. Thus, successful interventions in childhood and adolescence are needed to reduce the risks associated with overweight and obesity.

Overweight and obesity-related interventions often begin with the assessment of and overall risk status of participants^(7,8)^. Some methods of assessing body fat and composition include total body water, total body potassium, bioelectrical impedance analysis, dual-energy X-ray absorptiometry and body mass index (BMI)^(8)^. BMI (the ratio of weight in kilograms to height in metres squared) is the most common method of assessing obesity prevalence in population studies^(8,9)^. BMI can objectively be measured by trained researchers or self-reported by participants to estimate outcomes in health-related interventions^(8,10)^. To collect objective measurements, trained personnel are needed, as well as access to accurate and appropriate equipment^(11)^. Even though collecting objective measures of BMI tends to be most accurate, it may not be feasible in larger studies or programmes due to cost, time and available resources^(11,12)^. Self-reported measures, however, are generally practical, low cost, quick and easy to collect, and may provide benefits in sampling, recruitment and data collection, particularly for large populations^(9–11)^. Self-reported height and weight have been used to define BMI in college students and adults in instances where objectively measured values are not feasible^(13,14)^.

However, some research suggests that self-reported measurements are liable to biases that may arise from either individual bias in reporting behaviour or systematic differences in the type of survey used in the study^(9,15)^. Some sources of bias may include socio-economic status, body image perceptions and health-related behaviours such as physical inactivity and substance use^(16)^. In addition, gender may be a factor in differing reporting of height and weight. For example, females underreported their weight more than males in one study that examined self-reported height, weight and BMI in children and adolescents^(10)^. Pursey *et al.* also found that females underestimated their weight more than males, and that self-reported height was statistically different for both genders when examining young adults^(17)^. Zhou *et al.*^(18)^ examined the accuracy of self-reported weight, height and resultant BMI values in Chinese adolescents (aged 12–16) and observed wide discrepancies in self-report and objectively measured variables. These discrepancies were influenced by the area of residence, age and BMI category but not gender^(18)^. Regardless of potential biases, many studies reported that using self-reported measures were valid proxies for when collecting objective measures were not feasible^(9,10,19)^. Yet, some caution the discrepancies may impact outcomes and correction factors should be applied, when able^(11,12)^.

Self-reported data may provide an understanding of adolescent obesity, its correlates, precursors and impacts^(16)^. Though there is research on the relationship between self-reported and objectively measured height and weight in some adolescent populations, the evidence is not necessarily generalisable to the USA as most recent studies have been conducted outside of the USA or were conducted in one isolated location in the USA^(18–22)^. In addition, studies that were conducted in larger, more diverse samples were done in the early 2000s, warranting updated studies to explore whether trends of reporting measurements have changed^(23,24)^. Further research is needed to compare the accuracy of self-reported data with objectively measured height, weight and BMI in large adolescent populations^(25)^. Thus, the aims of the present study were to examine the accuracy of self-reported height, weight and resultant BMI values, and to describe gender differences in the level of agreement between objectively measured and self-reported values in a sample of high school students in different U.S. states.

## Methods

The present study was part of the Get Fruved project, a health and wellness initiative that aimed to prevent unwanted weight gain in college and high school students. During the high school development phase, researchers at four U.S. universities recruited and objectively measured height and weight of participants at five high schools. Each university recruited students in-person, through email correspondence and flyers. To be eligible, participants only had to be students of the participating high school. The study took place over 6 months to ensure a large sample size was obtained.

### Study/survey procedures

Participants reported their heights and weights prior to being measured by researchers. Once self-reported measurements were provided by the participant, objectively measured height and weight were collected as the gold standard reference for comparison. The Get Fruved project had a standard protocol for collecting anthropometric measurements, and lead trainers at each location trained researchers on this protocol^(26)^. Each researcher had to meet 80 % inter-rater reliability with the lead trainer during training sessions. Each measurement was taken twice and had to be within 0⋅2 kg and 0⋅2 cm for weight and height, respectively; otherwise, a third measurement was taken and the two measures within the specified range were entered. The mean of the two measurements was calculated for use in the study. The researchers entered all measurements into a secure platform, Qualtrics (Qualtrics, Provo, UT, USA). The technical error of measurement (TEM), the relative technical error of measurement (rTEM) and the coefficient of reliability (*R*) were calculated to assess the precision of the researchers that collected measurements. Height and weight revealed a TEM of 1⋅22 cm and 0⋅55 kg, respectively. The lower the TEM, the better the precision of the researcher^(27)^. The rTEM for height and weight were 0⋅73% and 0⋅82%, respectively. A rTEM less than 2% is considered acceptable^(27)^. The *R* for height and weight were 0⋅99 and 0⋅98, respectively. A value of over 0⋅95 is considered acceptable^(27)^. Precision of the researchers was deemed acceptable for this study. Prior to participating in the study, participants were provided with an assent form to read and sign. Only participants who assented for their data to be used and who had parental consent for their data to be used were included in analysis. This study was conducted according to the guidelines laid down in the Declaration of Helsinki, and all procedures involving human subjects were approved by the University of Tennessee Institutional Review Board (UTK IRB-14-09366 B-XP).

### Statistical analysis

Descriptive statistics were used to describe participant characteristics, such as age, gender, race and ethnicity, and state of residence. Continuous BMI scores were categorised (underweight, normal, overweight or obese) using BMI-for-age percentile, which was calculated using the Centers for Disease Control and Prevention's Children's BMI Group Calculator – Metric Version in Excel. Participants below the 5th percentile were categorised as underweight, between the 5th and 85th percentile were normal, 85th–95th percentile were overweight, and 95th or greater percentile were obese^(28)^. Two BMIs were calculated for each participant: one from self-reported height and weight and the other from the objectively measured height and weight by a trained researcher.

Pearson's correlation was used to understand the strength of the relationship between self-reported and objectively measured values. Even though Pearson's correlation is not the most appropriate method of correlation for this study, it was only included for comparison to other studies^(22,29)^. Lin's concordance correlation coefficient (CCC) is considered more appropriate and was used to measure precision and accuracy between self-reported and objectively measured BMI, height and weight^(30)^. Regression models were used to explore relationships of self-reported and objectively measured height, weight and continuous BMI scores with and without gender. Limits of agreement (LOA) analyses were conducted using the Bland–Altman method^(31)^. Bland–Altman plots were created for the overall sample, females and males to visually assess the agreement between BMI, height and weight. These visuals plot the difference of self-reported and objectively measured values against the mean of self-reported and objectively measured values to visually assess agreement^(31)^. Absolute mean differences (self-reported values minus objectively measured values) were calculated for each group's BMI, height and weight. The LOA was calculated by adding and subtracting the absolute mean difference's 95 % confidence interval to the absolute mean difference. In addition, paired sample *t*-tests were used to explore absolute mean differences between self-reported and objectively measured values.

An assessment of BMI category between self-reported and objectively measured values was conducted using weighted *κ* coefficients between gender, race and ethnicity, and state of residence^(32)^. Values are considered to have almost perfect agreement between 0⋅81 and 0⋅99, substantial agreement between 0⋅61 and 0⋅80, moderate agreement between 0⋅41 and 0⋅60, fair agreement between 0⋅21 and 0⋅40, slight agreement between 0⋅01 and 0⋅20, and less than chance agreement <0^(33)^. To assess how accurately participants in different BMI categories provide data to be placed in the correct category, frequency and percent of self-reported *v.* objectively measured BMI categories were presented. Considering each BMI category as the case of interest, the objectively measured BMI was used to classify participants, and sensitivity and specificity were calculated for each BMI category to assess the performance of self-reported measures *v.* objective measurements. Sensitivity was calculated by taking to the total cases that accurately reported the same category of their objective BMI category divided by the total number of objectively measured cases in that category^(34)^. Specificity was calculated by taking to the total number of cases that accurately reported not to be in the respective BMI category by the total number of cases that reported not to be in that category^(34)^. Sensitivity determines the proportion of correctly identified actual positives (cases), whereas specificity determines the proportion of correctly identified negatives (non-cases)^(18)^. To assess the accuracy of self-reporting height and weight to be classified into the correct BMI category, frequencies and percentages of those who underreported, accurately reported and overreported were presented for males, females and each BMI category. Pearson's *χ*^2^ test was used to assess discrepancies of expected BMI classification. All analyses were performed with Excel, R (version 4.0.0 for Windows, Vienna, Austria) and SPSS (Version 24.0. Armonk, NY), and the level of significance was *P <* 0⋅05.

## Results

A sample of 425 participants had both objectively measured and self-reported heights and weights collected. Five participants who had implausible values for either self-reported height and/or weight were removed from analysis. Since gender was a primary variable of interest, only participants that identified as either male or female were included in analysis. Eight participants were then removed for selecting their gender identity as ‘other,’ ‘choose not to answer’ or did not answer at all. This left a total sample of 412 participants.

Participants were almost evenly split by sex (51⋅0 % were female), most identified as non-Hispanic white (50⋅4 %) and the largest percentage of the sample was from the state of Florida (46⋅6 %) (Table 1). The mean age of participants was 16⋅3 (±7⋅1 sd) years, and most (70⋅8 %) participants were in the normal BMI category^(28)^. Each characteristic had substantial agreement (range 0⋅64–0⋅77)^(33)^. Participants from South Dakota had the least agreement among the demographic characteristics (*K* 0⋅64, *P <* 0⋅001) and individuals from Tennessee had the most agreement (*K* 0⋅77, *P <* 0⋅001).

**Table 1.** Characteristics of high school participants

	All (*n* 412)		Male (*n* 202)		Female (*n* 210)		Kappa coefficient*
All	412	100	–	–	–	–	0⋅71
Gender	*n*	%	*n*	%	*n*	%	
Female	–	–	–		210	51⋅0	0⋅73
Male	–	–	202	49⋅0	–	–	0⋅69
Race and ethnicity	*n*	%	*n*	%	*n*	%	
Hispanic/Latino	36	9⋅2	13	6⋅9	23	11⋅4	0⋅76
Non-Hispanic black	87	22⋅3	47	24⋅9	40	19⋅8	0⋅73
Non-Hispanic white	197	50⋅4	94	49⋅7	103	51⋅0	0⋅74
Other (including biracial)	71	18⋅2	35	18⋅5	36	17⋅8	0⋅67
State	*n*	%	*n*	%	*n*	%	
Tennessee	33	8⋅0	15	7⋅4	18	8⋅6	0⋅77
South Dakota	118	28⋅6	58	28⋅7	60	28⋅6	0⋅64
Florida	192	46⋅6	110	54⋅5	82	39⋅0	0⋅72
Kansas	69	16⋅7	19	9⋅4	50	23⋅8	0⋅73

Abbreviations: BMI: body mass index; sd: standard deviation; kg: kilograms; cm: centimeters.

* Weighted *κ* used. All coefficients are significant (*P* < 0⋅001).

The Pearson's correlations between self-reported and objectively measured BMI, height and weight were strong (*r* 0⋅75, 0⋅86 and 0⋅84, respectively)^(35)^. Lin's CCC between self-reported and objectively measured BMI, height and weight was highly concordant (*ρ*_c_ 0⋅86, 0⋅81 and 0⋅92, respectively)^(36)^. Regression analysis suggested that when predicting the objective height and weight using self-reported values, gender was insignificant in predicting height (*P =* 0⋅875) and was marginally significant for weight (*P =* 0⋅057). However, regression analysis found that when predicting objective BMI using self-reported values, gender is a significant factor (*P =* 0⋅010), and the interaction term between gender and self-reported BMI was also significant (*P =* 0⋅011). This indicated that when predicting objective BMI using self-reported values, the different gender groups will likely have different slopes and intercepts.

**Fig. 1.**
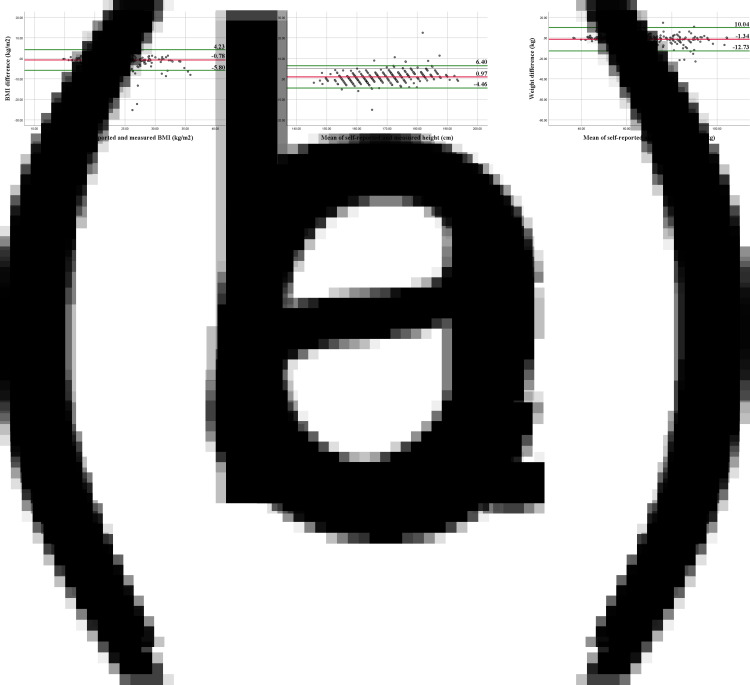
Bland–Altman plots of the difference *v.* the mean of self-reported and measured (a) body mass index (BMI), (b) height and (c) weight for the overall sample. Red line, mean difference between self-reported and measured data. Green lines, 95 % limits of agreement (1⋅96 sd).

**Fig. 2.**
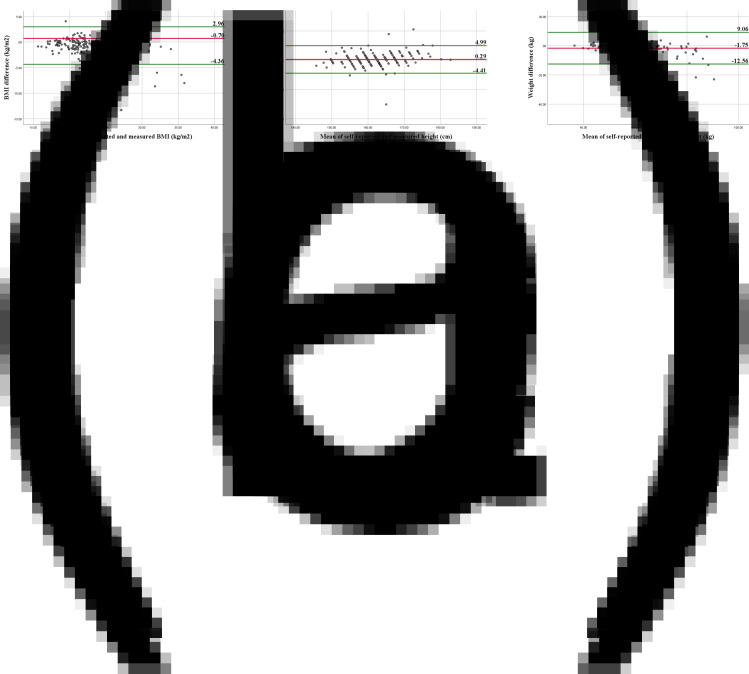
Bland–Altman plots of the difference *v*. the mean of self-reported and measured (a) body mass index (BMI), (b) height and (c) weight for female participants. Red line, mean difference between self-reported and measured data. Green lines, 95 % limits of agreement (1⋅96 sd).

**Fig. 3.**
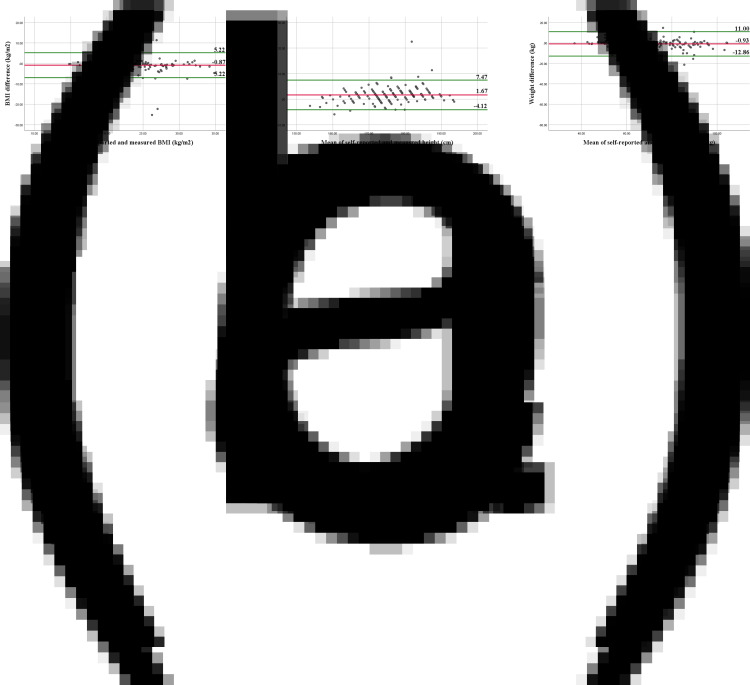
Bland–Altman plots of the difference *v*. the mean of self-reported and measured (a) body mass index (BMI), (b) height and (c) weight for male participants. Red line, mean difference between self-reported and measured data. Green lines, 95 % limits of agreement (1⋅96 sd).

The differences of self-reported and objectively measured values (for BMI, height and weight) were plotted against the mean of the two values for the overall sample, females and males (Figs. 1–3, respectively). The LOA, which is the 95 % confidence interval of the mean difference, was considered to have ‘good’ agreement if it was within 1 standard deviation (sd) of the mean of the objectively measured value, ‘fair’ agreement if within 2 sd and ‘poor’ agreement if within 3 sd^(10)^. The LOA of BMI was considered fair because it fell within 2 sd of the mean of objectively measured BMI for the overall sample, females and males (±8⋅67sd, ±8⋅37sd and ±8⋅98sd, respectively) (Table 2). For height, the LOA was considered good as each fell within 1 sd of the objectively measured mean of height for the overall sample, females and males (±9⋅62sd, ±6⋅68sd and ±7⋅70sd). The LOA for weight was considered to have good agreement for the overall sample and males due to falling within 1 sd of the objectively measured mean of weight (±14⋅98sd and ±15⋅86sd, respectively), but females had fair agreement due to falling within 2 sd of the objectively measured mean of weight (±23⋅38sd). Negative absolute mean differences indicated that BMI and weight were underreported in all groups, and the paired sample *t*-tests revealed that they all differed significantly (*P* < 0⋅001). For height, positive absolute mean differences revealed all groups overreported values, and paired sample *t*-tests revealed the overall sample and males differed significantly (*P* < 0⋅001), but females did not (*P* = 0⋅108).

**Table 2.** Limits of agreement for measured and self-reported BMI, height and weight

	Self-reported		Measured		Absolute mean difference^a^	Absolute mean difference LOA^b^	Agreement^c^	*P*-value*
	Mean	sd	Mean	sd				
					All (n 359)			
BMI (kg/m^2^)	22⋅38	3⋅78	23⋅16	4⋅34	−0⋅78	−5⋅80 to 4⋅23	Fair	<0⋅001
Height (cm)	169⋅61	10⋅61	168⋅67	9⋅62	0⋅97	−4⋅46 to 6⋅40	Good	<0⋅001
Weight (kg)	64⋅46	13⋅09	66⋅23	14⋅98	−1⋅34	−12⋅73 to 10⋅04	Good	<0⋅001
Females (*n* 181)								
BMI (kg/m^2^)	22⋅31	3⋅58	23⋅01	4⋅19	−0⋅70	−4⋅36 to 2⋅96	Fair	<0⋅001
Height (cm)	162⋅63	7⋅18	162⋅34	6⋅68	0⋅29	−4⋅41 to 4⋅99	Good	0⋅108
Weight (kg)	58⋅93	9⋅58	60⋅68	11⋅69	−1⋅75	12⋅56 to 9⋅06	Fair	<0⋅001
Males (*n* 178)								
BMI (kg/m^2^)	22⋅45	3⋅98	23⋅33	4⋅49	−0⋅87	−6⋅97 to 5⋅22	Fair	<0⋅001
Height (cm)	176⋅87	8⋅52	175⋅11	7⋅70	1⋅67	−4⋅12 to 7⋅47	Good	<0⋅001
Weight (kg)	70⋅18	13⋅77	71⋅88	15⋅86	−0⋅93	−12⋅86 to 11⋅00	Good	0⋅045

Abbreviations: sd: standard deviation; LOA: limits of agreement; BMI: body mass index; kg: kilograms; cm: centimeters.

a Absolute mean differences (self-reported − measured).

b LOA is 95 % confidence interval of the absolute mean difference.

c Agreement was considered ‘good’ if the LOA was within 1 sd of the measured mean, ‘fair’ if within 2 sd and ‘poor’ if within 3 sd.

* Paired *t*-test was calculated.

When continuous BMI values were categorised, most participants (94⋅0 %) that reported height and weight values to be in the normal BMI category were objectively measured for this category (Table 3). Sensitivity decreased as BMI increased from healthy to overweight to obese (94⋅0, 60⋅4 and 50⋅0 %, respectively); and specificity increased as BMI increased from healthy to overweight to obese (79⋅2, 91⋅1 and 99⋅0 %, respectively).

**Table 3.** Comparisons between self-reported and measured BMI categories

Self-reported BMI category	Measured BMI category								Self-reported BMI total
	Underweight		Normal		Overweight		Obese		
	*n*	%	*n*	%	*n*	%	*n*	%	*n*
Underweight	**7**	**77·8**	6	2⋅4	0	0⋅0	1	2⋅3	14
Normal	2	22⋅2	**237**	**94·0**	16	33⋅3	3	6⋅8	258
Overweight	0	0⋅0	9	3⋅6	**29**	**60·4**	18	40⋅9	56
Obese	0	0⋅0	0	0⋅0	3	6⋅3	**22**	**50·0**	25
Measured BMI total *n*	9		252		48		44		353
	%		%		%		%		
Sensitivity	77⋅8		94⋅0		60⋅4		50⋅0		
Specificity	98⋅0		79⋅2		91⋅1		99⋅0		

Abbreviations: BMI: body mass index. Bolded values indicate frequency and percentage of participants that accurately reported their BMI to be correctly categorised in the correct BMI category.

Most participants (83⋅6 %) accurately reported their height and weight enough to be categorised in the same BMI category as their objectively measured BMI (Table 4). Pearson's *χ*^2^ tests revealed that females more accurately reported BMI than males (87⋅3 *v.* 79⋅7 %, respectively). In addition, males had a higher percentage of overreporting BMI category than females (16⋅9 *v*. 8⋅3 %, respectively). Differences between gender and BMI categorisation were close to significant (*P =* 0⋅050). Regarding BMI category and reporting, Pearson's *χ*^2^ revealed significant reporting differences between BMI categories (*P* < 0⋅001). Those who were measured to be in the overweight or obese category had a higher than expected cell count for underreporting (33⋅3% and 50.0 % underreported, respectively), and those who measured to be in the normal weight category had a higher expected cell count for accurately reporting (94⋅0 % accurately reported). This indicated that those in the overweight or obese category were more likely to underreport their self-reported BMI to be placed in the incorrect BMI category.

**Table 4.** Accuracy of self-reported measures to be classified in the measured BMI category

	Underreported		Accurately reported		Overreported		*P*-value*
	*n*	%	*n*	%	*n*	%	
All	44	12⋅5	295	83⋅6	14	4	
Females	15	8⋅3	158	87⋅3	8	4⋅4	0⋅050
Males	29	16⋅9	137	79⋅7	6	3⋅5	
Underweight	–	–	7	77⋅8	2	22⋅2	<0⋅001
Normal weight	6	2⋅4	237	94⋅0	9	3⋅6	
Overweight	16	33⋅3	29	60⋅4	3	6⋅3	
Obese	22	50⋅0	22	50⋅0	–	–	

Abbreviations: BMI: body mass index.

* Pearson's *χ*^2^ between groups of variables and BMI classification accuracy.

## Discussion

Correlations of BMI, height and weight were strong and concordant for the overall sample. The LOA between self-reported and objectively measured BMI was fair for the sample and by each gender. Kappa was substantial for the overall sample and when measures were stratified by gender, race and ethnicity, and location. Most participants accurately reported their height and weight to be categorised in the correct BMI category. Gender differences occurred in reporting height and weight, and those who were objectively measured to be in the overweight or obese category were less accurate in self-reporting height and weight than their normal weight counterparts.

The overall sample had a strong correlation and concordance for BMI, height and weight. Though Pearson's correlation in the present study was lower than was found in another study which assessed the agreement of self-reported height and weight in adolescents^(10)^, correlations were similar to a study on emerging adults and slightly higher than pooled correlations found in a meta-analysis of fifteen studies^(36,37)^. Lin's CCC also fell in the ranges presented in the study with emerging adults^(36)^. Concordance was higher for weight and BMI, and lower in height, which was similar to another study on adolescents that used Lin's CCC to assess concordance^(22)^.

Height was overreported and weight was underreported, leading to slight underreporting for BMI in the overall sample. This finding is in agreement with other studies^(17,38,39)^. Visually, the Bland–Altman plot showed the higher the BMI, the less agreement among values. This was visually assessed as similar in other studies^(10,18,40)^. The overall sample's LOA were larger for weight and BMI, and smaller for height than a study conducted by Zhou *et al.*^(18)^ By using its study's specified criteria for the strength of agreement, Zhou *et al.* concluded that all three measures were unacceptable, especially BMI^(18)^. The present study used another *a priori* criteria for agreement presented by Yoshitake *et al.*^(10)^ In the study by Yoshitake, the authors concluded that BMI, height and weight were regarded as acceptable due to falling within 1 sd of the objectively measured mean^(10)^. In the present study, height and weight were considered to have good agreement, but BMI did not fall within the 1 sd for good agreement. Another study had similar overall strength of agreement for BMI, height and weight as the present study, and its authors also reported the values to have fair agreement^(17)^. When assessing weighted *κ* statistics for BMI categories, one study by Kee *et al.*^(19)^ had a *κ* of 0⋅76, which was only slightly higher than the overall sample's *κ* in this study (0⋅71), both indicating substantial agreement between self-reported values and BMI categorisation.

The regression models found gender differences in predicting BMI, which warranted further analyses. The present study found that both females and males significantly underreported weight, but only males significantly overreported height. This is contrary to an earlier study conducted in the USA by Brener *et al.*, which found that only female adolescents were more likely to underestimate their weight^(24)^, and another study by Pursey *et al.* which was conducted in Australia^(17)^, justifying conducting updated studies in the USA to measure agreement and accuracies of self-reported height and weight in adolescents. The findings of the present study also contradicted Pursey *et al.*'s finding that found differences in height between self-reported and objectively measured height were significant for both males and females^(17)^. In a literature review on self-reported and objectively measured comparison studies in adults, height was overreported for both sexes and underreported for weight and BMI^(38)^. The LOA analysis suggested that BMI for both males and females had only fair agreement, and height had good agreement. However, male weight had good agreement, but female weight had fair agreement. Two other studies in adolescents found similar mean differences and LOA for BMI, height and weight^(18,41)^. While they both concluded that agreement was fair on a population level, it was deemed unacceptable on an individual level^(18,41)^. When BMI was categorised, the present study found females to have higher agreement than males. This was different from a previous study conducted by Yoshitake *et al.* that found the opposite between genders^(10)^. The finding in the present study was supported by more females accurately reporting height and weight to be categorised in the accurate BMI category. This may be due to the overestimation of reported height by males previously reported. Discrepancies in height measurements impact BMI classification more than weight^(42)^. Many different factors may have led to males and females both underreporting their weight. In a study by Rasmussen *et al.*, females who underestimated weight had not been recently weighed, either at a doctor's office or did not weigh themselves^(20)^. In addition, the same study found that both males and females had low recall ability^(20)^.

When assessing differences in reporting between BMI category, the present study had similar findings to other studies^(12,21,22,38)^. Overweight and obese participants misreported height and weight more to be placed in incorrect BMI categories, where normal weight participants had high accuracy. A meta-analysis conducted by He *et al.* found that individuals who were overweight and obese were less accurate in BMI classification as well^(21)^. However, one study found a high agreement of BMI categorisation among overweight children^(10)^. Sensitivity analysis found that as BMI increased from normal to obese, sensitivity decreased, which supports the inaccuracies of BMI in overweight and obese individuals. A study in the USA found the sensitivity of obese adolescents ranged from 70⋅8 to 81⋅9 %, which was considerably higher than the 50⋅0 % found in this study^(22)^. Additionally, specificity analysis in the present study found that as BMI increased, specificity did as well, which was in accordance with expectations. Several studies suggested that weight underestimation may be associated with increased BMI in an adult population^(12,38,42)^. This was supported in our study which found high inaccuracies of BMI categorisation and decreased sensitivity as BMI increased.

When comparing *κ* statistics among participant characteristics, location provided the most variability in reliability between measures. South Dakota participants had the lowest agreement of BMI category, but Tennessee had the highest. In the study by Zhou *et al.*, the researchers found that the area of residence was a factor in BMI misclassification in participants, which supports this discrepancy^(18)^. Interestingly, a study conducted by Olfert *et al.* assessed the agreement of measurements in college students and found that participants from South Dakota had one of the highest agreement values over other states in the sample (which included states represented in this study)^(25)^. Why adolescents from South Dakota do not report height and weight as accurately as other U.S. states warrant further examination.

Previous studies that examined differences between self-reported and objectively measured BMI, height and weight, showed mixed results on whether self-reported measurements were an adequate proxy for objective measurements^(18,21)^. Findings seem to vary across location, which in one study found North America self-reported measures to be more biased than in Asia^(38)^. This study found lower agreement when using continuous values of BMI than categorizing the values into BMI categories (underweight, normal weight, overweight and obese). Thus, the authors caution future studies with U.S. adolescents against using continuous BMI variables over BMI categories, especially in smaller studies. This conclusion is consistent with other adolescent BMI agreement studies^(18,41)^. One way to mitigate the low agreement may be to implement a corrective factor to improve agreement between measures, which has been done in other comparison studies^(12,22)^.

Limitations of this study included not assessing factors that may have led to height and weight misreporting as other studies did^(16,20)^. One study found that body image perception and socio-economic status were predictors of misreporting^(16)^. In addition, though participants were from four states across the USA, it was not racial or ethnically diverse as the majority of students identified as non-Hispanic white. Therefore, results cannot be generalised to the U.S. adolescent population. Regardless, assessing participants from four states increases the strength of this study as BMI agreement levels were found to be different between states.

### 

#### Conclusion

Overall, a reasonable agreement between BMI classification in adolescents with objective and self-reported measures was found; however, further research is needed to explore regional differences in self-reported measurements. In addition, a more diverse sample should be utilised to make results more generalisable to the U.S. population. This study found greater agreement when height and weight were calculated to be placed in a BMI category than when used as a continuous variable; therefore, categorizing BMI is recommended for the adolescent population. Future research could focus on creating an algorithm to correct BMI misclassification to improve accuracy for self-reported data in adolescent studies.
